# Brucellosis Presenting with Pericarditis: Case Report and Literature Review

**DOI:** 10.1155/2013/796437

**Published:** 2013-01-17

**Authors:** Fábio Lopes Pedro, Fernanda Paula Franchini, Leonardo Muraro Wildner

**Affiliations:** ^1^Universidade Federal do Rio Grande do Sul, Porto Alegre, RS, Brazil; ^2^Hospital Universitário de Santa Maria, Santa Maria, RS, Brazil; ^3^Universidade Federal de Santa Maria, Santa Maria, RS, Brazil

## Abstract

Pericarditis is a rare manifestation during the course of brucellosis. This paper describes a case of pericarditis associated with brucellosis in a 31-year-old veterinary physician with a past medical history of testicular tumor and reviews the cases of pericarditis associated with brucellosis in medical English literature.

## 1. Introduction

Brucellosis is a zoonotic disease caused by Gram-negative bacteria from the genus *Brucella*. With a worldwide distribution [1, 2], it is considered hyperendemic in South America [3]. More than half a million new cases are diagnosed each year [1], and the prevalence rate exceeds 10 cases per 100,000 inhabitants in some countries [2]. 

The infection is transmitted to humans by animals, due to occupational exposure or ingestion of contaminated dairy products and meats [1]. With a wide spectrum of clinical manifestations [4], it can affect any organ or tissue in the human body [1]. The most commonly affected systems are the musculoskeletal, gastrointestinal, genitourinary, hematological, and respiratory systems [5]. 

Acute cases typically present with chills, fever, asthenia, sweating, weight loss, and back pain [5]. The most common findings on physical examination are hepatomegaly and splenomegaly [6]. Cardiovascular complications are rare, [2] with endocarditis being the most common form of cardiovascular system involvement [7]. 

The purpose of this paper is to report a case of brucellosis that presented with mild pericarditis during its course, not associated with endocarditis or myocarditis. 

## 2. Case Report

Male patient, Caucasian, 31 years old, from São Martinho da Serra, RS, Brazil. He resides with his wife and his mother. He had a past medical history of testicular tumor at age 24 and was cured after treatment. He had been asymptomatic for five years. 

On 07/01/2012, he started having fever (40.2°C). He sought medical treatment with the assistant oncologist, who requested additional tests to rule out tumor recurrence. A total abdominal ultrasonography showed hepatosplenomegaly and retroperitoneal lymph node enlargement. A transthoracic echocardiogram showed signs suggestive of acute pericarditis with an asymptomatic patient from the cardiologic point of view.

On the 15th day of symptoms, due to clinical and body temperature worsening, he sought the emergency care department of a local hospital, where he was treated by an infectologist. At this point, a detailed clinical history was obtained. He had sustained high fever, above 39°C, which did not improve with antipyretics used for the last five days (dipyrone 4 g/day and acetaminophen 3 g/day), accompanied by fetid and profuse night sweats (approximately four changes of clothing a night); asthenia; myalgia, especially in the dorsal region; mood alterations. The patient was a veterinarian who had been exposed to the premature delivery of bovines in the past six months. He denied intake of dairy products at home.

Considering the symptoms and the epidemiology of exposure to cattle, the hypothesis of a brucellosis diagnosis was raised. Laboratory assessment was carried out immediately. The patient was released to undergo home observation and to be reevaluated within 48 hours. In this assessment, the serology screening test (Rose Bengal test) for *Brucella* sp. IgM was positive, while the IgG fraction was negative. The patient had three blood cultures in progress. Empirical antimicrobial therapy was initiated for brucellosis with doxycycline (dose = 200 mg day) and rifampicin (dose = 900 mg/day).

He was maintained on outpatient assessment and reassessed on 07/22/12, when blood culture results were released, showing to be positive for Gram-negative bacilli. New serologic tests for brucellosis were requested. The IgM fraction remained positive, while there was seroconversion from negative to positive IgG antibody to brucellosis. Treatment was maintained with the above-mentioned doses.

Other general laboratory tests showed slight increase in transaminases and positive antinuclear antibodies (speckled pattern). For other test results, see Table 1.

The patient was asymptomatic after 10 days of treatment. On reassessment after 30 days, he showed improved overall health status with the presence of mild night sweats, increased appetite and return to work activities. He had been afebrile for more than three weeks. Treatment was maintained at the above-mentioned doses and was completed in 42 days.

After 60 days of symptom onset, the patient was asymptomatic and afebrile. He was discharged and received instructions in case of symptom recurrence.

## 3. Discussion

In spite of its worldwide distribution, the fact of being considered endemic in some locations [2], and because of its broad spectrum of clinical manifestations—therefore having many differential diagnoses, both infectious and noninfectious [8], brucellosis remains underdiagnosed and underreported [9]. 

Among the wide spectrum of clinical manifestations [4], cardiovascular manifestations, such as endocarditis, myocarditis, and pericarditis are rare [2, 10]. Of these, endocarditis is the most common, and the aortic valve is the most often affected site [10]. Endocarditis is also the most serious manifestation, contributing with more than 5% of the total rate of mortality by brucellosis [6]. When it occurs, the onset of myocarditis and pericarditis together is common, but its isolated appearance is extremely rare [2, 11]. 

After carrying out a search on MEDLINE database using the words “pericarditis” and “brucellosis,” 18 references were identified, dating from 1982 to 2011. Of these, only five papers contained reports of infections in humans with information in English on pericarditis associated with brucellosis, with the last cases having been reported in 2011. 

In a series of recent cases from a hospital located in Turkey, where the clinical manifestations were reviewed in 240 patients diagnosed with brucellosis, cardiac manifestations suggestive of pericarditis were identified in two cases (0.8%) [7]. In another series of cases including several hospitals, also located in Turkey, 1028 cases of brucellosis were retrospectively analyzed. Cardiac involvement was observed in seven patients (0.7%), all diagnosed with endocarditis [1]. 

The first two cases published in English language in 1985 reported the finding of isolated pericarditis and important pericardial effusion as the main clinical manifestation, both progressing to cardiac tamponade [12], different from what occurred in this reported case, where pericarditis was asymptomatic and the main clinical manifestations were fever and involvement of the general health status. 

On the other hand, and more similar to the present report, is a case reported in 2011, where a large-volume pericardial effusion was found at the transthoracic and transesophageal echocardiographies, which were performed to investigate endocarditis, as the patient had a systolic murmur on auscultation, although he was asymptomatic [10]. Our patient also underwent transthoracic echocardiography to rule out endocarditis, as part of investigation for fever of unknown origin. The possibility of endocarditis was excluded in our case, in spite of positive blood cultures, due to a combination of factors such as the absence of vegetations on echocardiography, the absence of a new heart murmur and a good clinical outcome after starting antibiotic therapy. 

We chose not to perform a pericardiocentesis, as the patient was asymptomatic from the cardiovascular point of view and due to the size of the pericardial effusion. 

The fact that the IgM serology for *Brucella* sp. was positive and there was seroconversion of IgG serology for *Brucella* sp., together with good clinical outcome after starting the antibiotic therapy corroborated the diagnosis.

Although the antibiotic regimen of choice for treatment of brucellosis is tetracycline plus streptomycin [5], the latter antibiotic is not released for treatment of brucellosis in Brazil. The regimen with doxycycline plus rifampicin is suited for oral treatment, when excluded skeletal complications such as discitis, meningitis, and endocarditis [5]. 

Considering that our patient was stable without requiring hospitalization, and showed no bone complications, meningitis, or endocarditis, we choose to use rifampicin plus doxycycline. 

## 4. Conclusion

Cardiac manifestations are uncommon in the course of brucellosis, with the most common being endocarditis. Pericardial effusion may occur even as an isolated cardiovascular symptom and may manifest both symptomatically and asymptomatically. In areas where brucellosis is endemic, it is important to consider the fact that pericarditis can occur without symptoms due to the possibility of pericardial tamponade and sudden death. Furthermore, it is important to consider brucellosis as a differential diagnosis in cases of acute pericarditis.

## Figures and Tables

**Table 1:** Laboratory assessment.

Variable/(unit)/(reference values)	Before symptom onset	Symptom onset	After 15 days
Hemoglobin (g/dL) ()	13.6	12.6	
Hematocrit (%) (37–47)	40.5	39.5	
Leukocytes (per mm^3^) (4,000–10,000)	4.500	4.300	
Neutrophils (per mm^3^) (2,000–8,000)	2.520	1.720	
Lymphocytes (per mm^3^) (1,000–4,000)	1.170	1.935	
Platelets (per mm^3^) (150–400, 10^3^)	185.000	181.000	
Alanine aminotransferase (U/L) (<59)	102		95
Aspartate aminotransferase (U/L) (<72)	51		50
Gamma-glutamyltransferase (U/L) (<73)	162	226	
Lactic dehydrogenase (U/L) (313–618)	660	828	
Chorionic gonadotropin (mIU/mL) (<5)	<1		
Alpha-fetoprotein (IU/mL) (<5)		1.7	
Erythrocyte sedimentation rate (mm) (<15)		15	
HIV I/II antibodies			Nonreagent
Epstein Barr antibodies IgM (U/mL) (<20)			10
Epstein Barr antibodies IgG (U/mL) (<20)			288
Cytomegalovirus antibodies IgM			Nonreagent
Cytomegalovirus antibodies IgG			Reagent
Toxoplasmosis antibodies IgM			Nonreagent
Toxoplasmosis antibodies IgG			Nonreagent
Hepatitis B—surface antigen			Negative
Hepatitis C—anti HCV antibodies			Nonreagent
Antinuclear antibody			1/80
Rheumatoid factor (IU/mL) (<14)			14.45
